# Graft Versus Host Disease Associated with Immune Checkpoint Inhibitors: A Pharmacovigilance Study and Systematic Literature Review

**DOI:** 10.3389/fphar.2020.619649

**Published:** 2021-02-05

**Authors:** Lee S. Nguyen, Lisa Raia, Bénédicte Lebrun-Vignes, Joe-Elie Salem

**Affiliations:** ^1^ CMC Ambroise Paré, Research and Innovation—RICAP, Neuilly-sur-Seine, France; ^2^ Sorbonne Université, Clinical Investigations Center Paris-Est, AP.HP.6 Pitie-Salpetriere University Hospital, INSERM, Paris, France; ^3^ Intensive Care Medicine Department, AP.HP.Centre Cochin University Hospital, Paris, France; ^4^ Créteil Paris-Est University, EpiderMe, Creteil, France; ^5^ Department of Medicine, Cardio-Oncology Program, Vanderbilt University Medical Center, Nashville, TN, United States

**Keywords:** immunotherapy, pharmacovigilance, vigibase®, graft-versus-host disease, adverse (side) effects

## Abstract

**Background:** In patients with allogenic hematopoietic stem cell transplantation (allo-HSCT), immune-checkpoint inhibitors (ICI) are used to treat malignancy recurrence. However, ICI are also associated with graft vs. host disease (GVHD). In this pharmacovigilance analysis, we aimed to characterize cases of GVHD associated with ICI, drawn from the World Health Organization pharmacovigilance database, VigiBase®, and from literature.

**Methods:** We performed VigiBase® query of cases of GVHD associated with ICI. These cases were combined with those of literature, not reported in VigiBase®. The Bayesian estimate of disproportionality analysis, the information component, was considered significant if its 95% credibility interval lower bound was positive; denoting a significant association between GVHD and the suspected ICI. Time to onset between ICI and GVHD onset and subsequent mortality were assessed.

**Results:** Disproportionality analysis yielded 93 cases of GVHD associated with ICI (61.8% men, median age 38 [interquartile range = 27; 50] years). Cases were mostly associated with nivolumab (53/93, 57.0%), pembrolizumab (23/93, 24.7%) and ipilimumab (12/93, 12.9%) monotherapies. GVHD events occurred after 1 [1; 5.5] injection of ICI, with a time to onset of 35 [IQR = 14; 176] days. Immediate subsequent mortality after GVHD was 24/93, 25.8%. There was no significant difference in mortality depending on the molecule (*p* = 0.41) or the combination regimen (combined vs. monotherapy, *p* = 0.60). Previous history of GVHD was present in 11/18, 61.1% in cases reported in literature.

**Conclusion:** In this worldwide pharmacovigilance study, disproportionality yielded significant association between GVHD and ICI, with subsequent mortality of 25.8%. Previous history of GVHD was reported in more than half of cases.

**Clinicaltrials.gov identifier:** NCT03492242

## Background

Immune checkpoint inhibitors (ICI) may be used to treat aggressive hematologic malignancies, either in refractory or relapsed lymphoma frequently before being treated by allogeneic hematopoietic stem cell transplantation (allo-HSCT) or in relapse after allo-HSCT. Indeed, in both situations, relapse mechanisms include immune escape by the tumor, T-cell anergy, down-regulation of regulatory T cells and activation of immune checkpoints (Barrett and Battiwalla, 2010). They include drugs targeting programmed death-1 receptor (anti-PD-1), its ligand (anti-PD-L1) and cytotoxic T lymphocyte antigen-4 (anti-CTLA-4).

Like other therapies used in these indications (i.e., donor lymphocytes infusion, chemotherapy, immunotherapy, CAR T cell therapy), the aim of ICI is to enhance the immune system so that it may be effective against malignancy, by restoring T-cell function, activating lymphocytes and inducing a sustainable graft-versus-tumor (GVT) effect.

However, ICI are associated with immune-related adverse events, secondary to the over-activation of the immune system, which may, in turn, cause auto-immune-like complications, including graft-versus-host disease (GVHD). GVHD is serious adverse event in patients with allo-HSCT, initially described as an aggravated manifestation of regular inflammation, in which, donor lymphocytes interact with recipient antigens which may cause multi-organ dysfunction (Ramachandran et al., 2019).

Previously, cohort studies showed that ICI used before allo-HSCT were associated with an incidence of GVHD varying from 41 to 56% (Merryman et al., 2017; Ijaz et al., 2019). When used after allo-HSCT, the incidence depended whether there was a history of previous GVHD (55%) or if it was the first episode (30%) (Haverkos et al., 2017; Herbaux et al., 2018).

To comfort these results yielded from cohort studies; in the present work, we used disproportionality methodology to present characteristics of GVHD following ICI administration. This method is based on a large pharmacovigilance database, VigiBase®, which collects worldwide reports of drug-related adverse events in the World Health Organization network (Lindquist 2008). In addition, whenever possible, we combined the individual reports yielded from VigiBase® to de-duplicated cases reported in the literature to expand the cohort.

## Methods

### Study Design

This work combines a worldwide pharmacovigilance observational case-control cross-sectional study focusing on GVHD related to the usage of ICI, and a systematic literature case report analysis. The pharmacovigilance part relies on VigiBase®, a database encompassing 22 million individual case safety reports (ICSR) received worldwide (Lindquist 2008). It is freely accessible upon request, increasing possibilities for external validation (“VigiAccess.” from http://www.vigiaccess.org/). ICSRs include administrative information (country, type of report, qualification of reporter), patient data (age, sex), date of onset of reaction(s) and nature of the outcome using the latest version of MedDRA (Medical Dictionary for Regulatory Activities) terms (currently v22.1). Drug(s) involved (name, drug start and stop dates, indication, dose) are also indicated.

We searched for cases flagged with MedDRA preferred terms (PT) reflecting GVHD (see below), and associated with ICI molecules (nivolumab, pembrolizumab, atezolizumab, durvalumab, avelumab, ipilimumab, tremelimumab and cemiplimab) from VigiBase® creation through January 05, 2020.

This work is ancillary to the Immune CHeckpoint Inhibitors Monitoring of Adverse Drug ReAction (CHIMeRA) registry (clinicaltrials.gov registry number NCT03492242).

### Disproportionality Analyses

To assess whether a GVHD adverse event was associated with an ICI molecule in VigiBase®, we used disproportionality analysis (also known as case–non-case analysis) methodology. Briefly, the estimate of disproportionality analysis can be calculated by the information component (IC) for which IC_025_ is the lower end of its 95% credibility interval. A positive IC_025_ is statistically significant (Bate et al., 1998; Norén et al., 2013). The IC calculation was based on the number of GVHD reported with each ICI molecule, vs. all ADR with all medicines reported in VigiBase®. Like others, our research group previously used this method and database to describe the spectrum of cardiovascular diseases and other immune-related adverse events associated with ICI (Salem et al., 2018; Nguyen et al., 2020).

Calculation of the IC using a Bayesian confidence propagation neural network was developed and validated by the Uppsala Monitoring Center as a flexible, automated indicator value for disproportionate reporting that compares observed and expected drug–ADR associations to find new drug–ADR signals with identification of probability difference from the background data (full database) (Bate et al., 1998). Probabilistic reasoning in intelligent systems (information theory) has proved to be effective for the management of large datasets, is robust in handling incomplete data, and can be used with complex variables. The information theory tool is ideal for finding drug–ADR combinations with other variables that are highly associated compared with the generality of the stored data (Bate, Lindquist et al., 1998). Several examples of validation with the IC exist, showing the power of the technique to find signals sooner after drug approval than by a regulatory agency (e.g., an association between captopril and coughing), and to avoid false positives, whereby an association between a common drug and a common ADR occurs in the database only because the drug is widely used and the ADR is frequently reported (Bate, Lindquist et al., 1998; Norén, Hopstadius et al., 2013). Furthermore, our group recently published several studies using VigiBase® and disproportional reporting calculation to characterize and identify new drug-ADR associated signals, which were subsequently corroborated by preclinical mechanistic studies or prospective cohorts (Salem et al., 2018; Salem et al., 2019a; Salem et al., 2019b; Salem et al., 2019c).

The statistical formula is as follows:

$$\text{IC}=\log2\left[\frac{\left(N_{\text{observed}}+0.5\right)}{\left(N_{\text{expected}}+0.5\right)}\right]$$

where

$$N_{\text{expected}}=\left[\frac{\left(N_{\text{drug}}\times N_{\text{effect}}\right)}{N_{\text{total}}}\right]$$

*N*_expected_ is the number of case reports expected for the drug–ADR combination.

*N*_observed_ is the actual number of case reports for the drug–ADR combination.

*N*_drug_ is the number of case reports for the drug, regardless of ADR.

*N*_effect_ is the number of case reports for the ADR, regardless of drug.

*N*_total_ is the total number of case reports in the database.

IC_025_ is the lower end of a 95% credibility interval for the IC.

A positive IC_025_ value (>0) is the traditional threshold deemed statistically significant. IC_025_ values have only been validated for comparison of drug-specific ADR vs. the full database and cannot be used to compare disproportionate reporting among different ICI regimens. All patients were included in these analyses.

List of all MedDRA preferred terms (PT) related to GVHD, used in VigiBase® query.

Acute graft vs. host disease (PT), Acute graft vs. host disease in intestine (PT), Acute graft vs. host disease in liver (PT), Acute graft vs. host disease in skin (PT), Chronic graft vs. host disease (PT), Chronic graft vs. host disease in intestine (PT), Chronic graft vs. host disease in liver (PT), Chronic graft vs. host disease in skin (PT), Graft vs. host disease (PT), Graft vs. host disease in eye (PT), Graft vs. host disease in gastrointestinal tract (PT), Graft vs. host disease in liver (PT), Graft vs. host disease in lung (PT), Graft vs. host disease in skin (PT).

### Literature Review

A systematic literature review of all published cases of GVHD associated with ICI was performed, spanning from January 1st, 2010 to February 1st, 2020, on MEDLINE using PubMed® search engine. The methodological search strategy included keywords associated with Medical Subject Headings (MeSH) terms related to ICI and GVHD: “immune checkpoints inhibitors” OR (“immune” AND “checkpoints”) OR (“checkpoint” AND “inhibitor”) OR “nivolumab” OR “pembrolizumab” OR “ipilimumab” OR “cemiplimab” OR “avelumab” OR “durvalumab” OR “atezolizumab” OR “PD-1 blockade”) AND (“graft vs. host disease” OR (“graft vs. host” AND “disease”) AND (“Allogeneic stem cell transplantation” OR (“allogeneic” AND “stem cell transplant”). From this search, 715 articles were found which contained any of these terms, but the combination of these terms in the format of case reports or case series after adequate filtering yielded 22 cases.

### Statistics

Continuous variables are presented as median [interquartile-range] and categorical variables as number (percentage), with relevant comparison tests, accounting for data distribution. GraphPad Prism v6.0 (GraphPad Software, California, United States) was used for statistics and figures, except Figure 2 drawn using R software (R project, worldwide community project).

**FIGURE 1:**
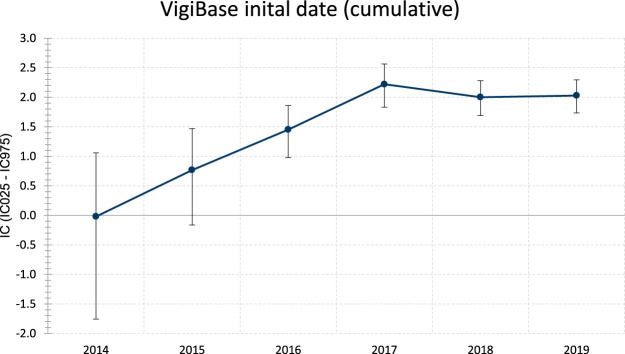
Evolution of information component (IC) over time of ICI associated GVHD (thru December 2019). Whiskers represent the 95% credibility interval lower and upper bounds (respectively IC_025_ and IC_975_). IC is considered significant when IC_025_ is >0.Statistics: IC = log2 [(*N*_observed_ + 0.5)/(*N*_expected_ + 0.5)], where *N*_expected_ = (*N*_drug_ × *N*_effect_)/*N*_total_, with *N*_expected_ being the number of ICSRs expected for the drug-ADR combination; N_observed_ being the actual number of ICSRs observed for the drug-ADR combination; *N*_drug_ being the number of ICSRs for the drug, regardless of ADR; *N*_effect_ being the number of ICSRs for the ADR, regardless of the drug; and *N*_total_ being the total number of ICSRs in the database. ADR = adverse drug reaction; ICSR = individual case safety report.

**FIGURE 2:**
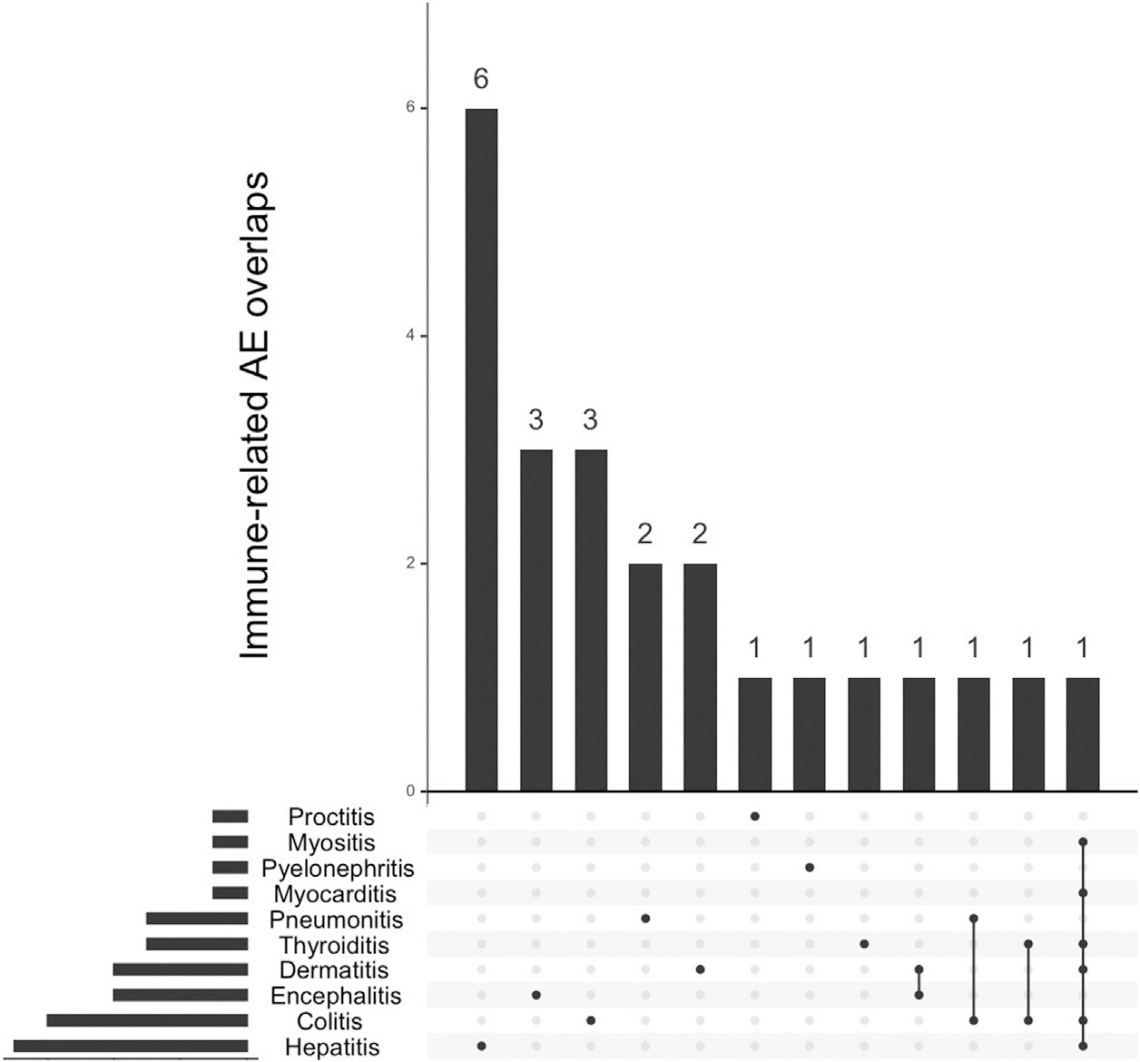
Overlap between concurrent immune-related adverse events (IrAE) with graft vs. host disease events associated with immune-checkpoint inhibitors (n = 23).

## Results

Overall, we included 93 deduplicated cases (91 reported in VigiBase® and 22 in literature, among which 20 were reported in VigiBase® that we accounted for). In VigiBase®, disproportionality analysis yielded significant association between GVHD events and ICI molecules, with an IC = 2.0 and IC_025_ = 1.7 (see Figure 1). The first reported case was with ipilimumab in 2014. The most represented drug-ADR association was nivolumab with GVHD (n = 33, IC = 2.4, IC_025_ = 1.9). The most common ICI indications reported were Hodgkin disease in 47/81, 58.0% and non-Hodgkin lymphoma in 14/81, 17.3%. ICI were prescribed in monotherapy, in 88/93, 94.6% (as compared to combination therapy involving several ICI). There was no relevant information regarding GVHD grade in VigiBase®.

In the aggregated dataset of 93 cases, men represented 42/68, 61.8% of cases (Table 1). Median age was 38 [interquartile range (IQR) = 27; 50] years. Events occurred after a median of 1 [IQR = 1; 5.5] ICI injection. Median time to onset of GVHD after first ICI injection was 35 [IQR = 14; 176] days (data available in 19/93, 20.4%).

**TABLE 1:** Reports of graft vs. host disease (GVHD) associated with immune checkpoint inhibitors (ICI) aggregated from VigiBase® (thru January 05, 2020) and systematic literature search (thru February 2020).

	n/N (%)	Data available, n (%)
Reporting region		All
United States	50/93 (53.8)	
Europe	23/93 (24.7)	
Australia	7/93 (7.5)	
Asia	13/93 (14.0)	
Reporting year		All
2019	32/93 (34.4)	
2018	19/93 (20.4)	
2017	29/93 (31.2)	
2016	9/93 (9.7)	
2015	3/93 (3.2)	
2014	1/93 (1.1)	
Reporters		89/93 (95.7)
Healthcare professional	82/89 (92.1)	
Non-healthcare professional	7/89 (7.9)	
Reports in the course of clinical studies	11/93 (11.8%)	All
Sex		68/93 (73.1)
Men	42/68 (61.8)	
Women	26/68 (38.2)	
Age at onset, mean ± SD, years	39.03 ± 16.25	61/93 (65.6)
(min-max)	(3–74)	
Death	24/93 (25.8%)	All
Suspected drugs		All
Only ICI	69/93 (74.2)	
ICI +1 other drug	11/93 (11.8)	
ICI + ≥2 other drugs	13/93 (14.0)	
Drugs		All
Monotherapy with anti PD-1/PD-L1		
Nivolumab	53/93 (57.0)	
Pembrolizumab	23/93 (24.7)	
Monotherapy with anti CTLA-4		
Ipilimumab	12/93 (12.9)	
Combination therapy		
Nivolumab + Ipilimumab	5/93 (5.4)	
Indications for ICI		81/93 (87.1)
Hodgkin disease	47/81 (58.0%)	
Non-hodgkin lymphoma	14/81 (17.3%)	
Other	20/81 (24.7%)	
Timing of ICI administration		22/93 (23.7)
Before allo-HCST	5/22 (22.7)	
After allo-HCST	17/22 (77.3)	
Time between first dose of ICI and GVHD onset, days:		19/93 (20.4)
Median; [IQR]	35 [14–176]	
(min-max)	(3–240)	
Type of graft		17/93 (18.3)
Matched related donor	12/17 (70.6)	
Matched unrelated donor	4/17 (23.5)	
Cord blood	1/17 (5.9)	
Previous GVHD		18/93 (19.4)
Yes	11/18 (61.1)	
No	7/18 (38.9)	

allo-HSCT: allogenic hematopoietic cell transplantation; IQR: interquartile range; PD-1: programmed cell death protein 1; PD-L1: Programmed death-ligand 1; CTLA4: cytotoxic T-lymphocyte-associated protein 4.

Overall mortality associated with ICI-associated GVHD was 24/93, 25.8%. This mortality was that reported in VigiBase® and implies a very short follow-up after the episode (less than a month). Therapy regimen (combination vs. monotherapy) did not affect subsequent mortality (respectively, 22/88, 25.0% vs. 2/5, 40.0%, *p* = 0.60). Mortality was not affected by the type of molecule (*p* = 0.41). Immune-related adverse events were co-reported in 23/93, 24.7%, mostly with hepatitis in 6/23, 26.1%.

In case reports extracted from literature (Singh et al., 2016; Boekstegers et al., 2017; Braun et al., 2017; Haverkos et al., 2017; Klobuch et al., 2017; Onizuka et al., 2017; Herbaux et al., 2018; Charles et al., 2019; Kim et al., 2019; Minson et al., 2019), when mentioned, ICI was indicated for disease relapse after allo-HSCT in 17/22, 77.3%. In one case of nivolumab-associated GVHD, biopsy showed PD-L1 expression in skin, liver and muscular tissues (those with active GVHD) (Charles et al., 2019).

Finally, we observed 11/18, 61.1% of patients had prior history of GVHD before ICI administration, although this information was lacking in 75/93, 80.6% of reports.

## Discussion

In this work, we analyzed GVHD related to ICI, by combining a systematic disproportionality analysis relying on the WHO pharmacovigilance database, VigiBase®; and case-reports drawn from literature, amounting a total of 93 cases to further describe risk factors of GVHD and prognosis.

In the cases we reviewed, ICI were mostly indicated for disease relapse after allo-HSCT, aiming at restauration of T-cell function and appropriate graft-versus-tumor effect (Godfrey et al., 2017). Whether previous GVHD is an additive or synergistic risk factor of GVHD remains to be explored, however better characterization of GVHD events may benefit from adding any prior history of GVHD, which remains the risk factor most associated with this event. Previous reports confirmed that history of GVHD was a risk factor, even for GVHD related to ICI after allo-HSCT (12/17, 70.6% vs. 5/17, 29.4%) (Haverkos et al., 2017).

While VigiBase® cannot be used to compute true incidence of adverse serious events, in this worldwide pharmacovigilance database analysis, we confirmed a significant association between GVHD and ICI administration. Time to onset was 35 days after first treatment, however, residual effects of ICI, due to a long half-life and extended pharmacodynamic effects, even substantially beyond its administration prior to allo-HSCT (Geraud et al., 2021). Indeed, expression of PD-1 on T cells was found significantly decreased up to 6 months after allo-HSCT, despite a last dose of ICI more than a month prior (Merryman et al., 2017). In patients not treated by ICI, PD-1 expression after allo-HSCT was also associated with increased risk of mortality (Schade et al., 2016).

Literature search yielded in one case of nivolumab-associated GVHD, a PD-L1 expression in skin, liver and muscular tissues (those with active GVHD) (Charles et al., 2019). As of yet, PD-L1 expression has not been described in regular GVHD reports (Ramachandran et al., 2019). Crosstalk between PD-1/PD-L1 and CTLA4 may play an important role in GVHD mechanisms. Indeed, while ICI may induce GVHD, reversion by abatacept was described (Nahas et al., 2018).

Interestingly, we found similar results as those found in the largest dataset to date, of GVHD due to ICI, aggregating 283 cases from several studies albeit with a reported overall mortality after GVHD of 11%, lower than that observed in our study. There were 107 cases occurring prior to allo-HSCT, and 176 after, akin to the results we observed in our dataset (Ijaz et al., 2019).

Our findings support the fact that ICI use in patients with allo-HSCT needs to be carefully monitored, as these patients are at high risk of developing GVHD. Regarding event prediction, risk factors of developing immune-related adverse events are currently under investigation (Shankar et al., 2020), and biomarkers involved in the mTOR pathway have been suggested (Esfahani et al., 2019).

We acknowledge several limitations to the present work. First, retrospective pharmacovigilance analyses present inherent intrinsic limitations; while disproportionality reporting used here (IC_025_) have been demonstrated to accurately to identify signals of cardiovascular adverse events associated with anti-cancer drugs in various settings (Salem et al., 2018; Salem et al., 2019b), risk of false signals remain possible. Reporting and publication bias need to be addressed: in most cases, details on patients such as previous history of GVHD were not mentioned in VigiBase® making it hard to assess its weight into the added risk of developing GVHD after ICI administration. Similarly, indications for ICI treatment or previous HSCT were not always clearly stated, making it hard to categorize these events. Cotreatments used to treat lymphoma relapse were not always stated, which makes the assertion of cumulative risk of GVHD difficult, as previous other treatments used in these indications may also be associated with GVHD, such as donor lymphocytes infusions. Finally, these analyses do not allow the computation of true incidence, as by essence, disproportionality analyses report relative incidences. True incidence would require a denominator which would include all worldwide prescriptions of ICI in the study period. Yet, our observations confirm several cohort studies and we hope these findings may help oncologists, hematologists and pharmacovigilance specialists to better interact in order to improve reporting and management of GVHD related to ICI intake.

## Conclusion

This worldwide pharmacovigilance database analysis confirmed a significant association between GVHD and ICI.

## Data Availability Statement

Publicly available datasets were analyzed in this study. This data can be found here: http://www.vigiaccess.org.

## Disclaimer

The supplied data from VigiBase come from various sources. The likelihood of a causal relationship is not the same in all reports. The information does not represent the opinion of WHO. The authors have nothing to disclose related to this study. The data, analytic methods, and study materials are available to other researchers for purposes of reproducing the results or replicating the procedure at http://www.vigiaccess.org/.

## Author Contributions

LN and LR equally contributed to the manuscript. They wrote the draft, performed analyses, reviewed literature. BL-V provided VigiBase data and provided critical review to the manuscript. J-ES supervised the project, performed analyses and provided critical review to the manuscript.

## Conflict of Interest

J-ES has participated to BMS advisory boards.

The remaining authors declare that the research was conducted in the absence of any commercial or financial relationships that could be construed as a potential conflict of interest.
